# Circadian immune system in solid organ transplantation: a review article

**DOI:** 10.3389/fimmu.2025.1556057

**Published:** 2025-03-03

**Authors:** Wai Yen Yim, Chenghao Li, Fuqiang Tong, Jincheng Hou, Yuqi Chen, Zongtao Liu, Zihao Wang, Bingchuan Geng, Yixuan Wang, Nianguo Dong

**Affiliations:** ^1^ Department of Cardiovascular Surgery, Union Hospital, Tongji Medical College, Huazhong University of Science and Technology, Wuhan, China; ^2^ Key Laboratory of Organ Transplantation, Chinese Academy of Medical Sciences, Wuhan, China

**Keywords:** circadian rhythm, chronotherapy, transplantation, transplant immunology, antigen presentation, time-of-day, transplant surgery

## Abstract

The innate and adaptive immune systems are intricately regulated by the circadian clock machinery. Recent clinical investigations have shed light on the influence of timing in organ procurement and transplantation on graft survival. In this review, we explore various mechanisms of immunological functions associated with the steps involved in organ transplantation, spanning from surgical harvesting to reperfusion and linking to the circadian rhythm. A deeper understanding of these processes has the potential to extend the principles of chrono-immunotherapy to the realm of organ transplantation, with the aim of enhancing graft durability and improving patient outcomes. This review concludes with some perspectives on future directions in this exciting and still evolving field of research.

## 1 Introduction

Organ transplantation stands as the definitive treatment for end-stage organ failure. Despite its remarkable efficacy, a pervasive challenge persists in the field—the shortage of donor organs and the efficacy of immunosuppressive regimens (1, 2). Strategies to maximize graft utilization and mitigate post-transplantation adverse effects include optimizing organ allocation systems, donor management and selection criteria, and protecting organs during retrieval and ischemia-reperfusion, have been devised (3, 4).

The circadian rhythm has garnered significant attention due to the pronounced time-of-day dependence exhibited by our immune system (5, 6). Evidence suggests that the efficacy of therapeutic interventions, such as (immune checkpoint inhibitors, sepsis strategies, vaccinations, and anti-inflammatory therapies), is influenced by circadian timing (7–9). Despite the mechanistic similarities shared by these therapeutic approaches, the role of circadian rhythm in organ transplantation has yet to be fully explored in clinical practice.

In this review, we aim to consolidate existing literature pertaining to the timing of donor graft retrieval, surgical reperfusion, and their clinical implications. Furthermore, we seek to elucidate the fundamental concepts underlying the molecular mechanisms of circadian clocks and their influence on the immune system, particularly in the context of transplant immunology. Specifically, we will delve into the role of circadian rhythms in modulating innate immune responses, the dynamics of passenger leukocytes, and the induction of immune tolerance in transplantation settings.

## 2 Time (hour) of transplantation

Numerous studies have investigated the timing of surgical procedures, including donor graft clamping (10–12) or de-clamping time (13, 14), as well as the impact of day-night effects or working/after-work hours (15–31) on recipient outcomes. In the following section, we provide a summary of the main clinical findings (**Table 1**) from studies examining various aspects of timing in organ transplantation, including donor graft retrieval, surgical reperfusion, and surgery start time.

**Table 1:** Summary of retrospective studies using donor clamp/de-clamping and first skin-incision time (hour) to compare transplant outcome.

Organ	Study, n	Grouping time	Clinical outcomes	Ref. (et. al.)
Grouping by using donor clamping time				
Heart	UNOS heart,n 31713	12AM-12PM vs 12PM-12AM	12AM-12PM showed significant increase long-term all-cause survival, 5PM-10PM had highest all-cause mortality.	Gouche DA (10)
Heart	Single,n = 399	12AM-12PM vs 12PM-12AM	12AM-12PM showed significant increase long-term allograft survival with decrease rejection rates. Insignificant difference in survival using de-clamping time.	Yim WY (11)
Kidney	EKiTE registry,n= 5026	1. 12AM-12PM vs 12PM-12AM2. 6PM-12AM vs 12AM-6PM	12AM-12PM were more protective towards delayed graft function.12AM-6PM were more protective towards graft failure. Insignificant difference in survival using de-clamping time.	Ville S (12)
Grouping by using donor de-clamping time				
Lungs	Single,n = 563	4AM-8AM vs 8AM-4AM	Lung re-perfused during high-risk window (4AM-8AM) had increased risk for PGD.	Cunningham PS (14)
Kidney	Cristal registry,n = 10,291	Night (6PM-10AM) vs Day (10AM-6PM)	Daytime associated with improved 1- and 3-year survival and lower incidence of death by graft failure at 1 year. Insignificant difference in survival using clamp time.	Montaigne D (13)
Grouping by using recipient first skin-incision time				
Heart and Lungs	UNOS heart,n = 16573;Lungs,n = 10545	Day (7AM-7PM) vs Night (7PM-7AM)	1. Insignificant difference in survival up to 1 year in heart transplant;2. Increased adjusted mortality at 90 days in lung transplant	George TJ (15)
Heart	Single,n = 329	Working (7:30AM-5:00PM) vs after (5:00PM-7:30AM)	Insignificant difference in early complications and survival up to 9 years.	Nishida H (17)
	Single,n = 235	4:00AM-11:59AM vs 12:00PM-07:59PM vs 8:00PM-03:59AM	Insignificant difference in short-term survival and peri-operative complications.	Immohr MB (22)
Lungs	Single,n = 740	Day (5AM-6PM) vs Night (6PM-5AM)	Nighttime associated with higher risk for major post-op adverse event, decreased overall survival and bronchiolitis obliterans syndrome-free at 5 years.	Yang Z (19)
Liver	UNOS,n = 94768	Day (7AM-7PM) vs Night (7PM-7AM)	Insignificant difference in survival at 1 year.	Orman ES (23)
	UNOS,n = 45980	Day (7AM-9PM) vs Night (10PM-6AM)	Insignificant difference in graft and patient survival.	Thuluvath PJ (24)
	Single,n = 578	Day (3AM-3PM) vs Night (3PM-3AM)	1. Insignificant difference in survival between group at 5 years.2. Higher risk for any postoperative complication at 3PM-6PM compared to 6AM-9AM group.	Lonze BE (16)
	Single,n = 350	Day (8AM-6PM) vs Night (6PM-8AM)	Insignificant difference in survival at 1 year.	Becker F (18)
	UK transplant, n = 8816	Day (7AM-7PM) vs Night (7PM-7AM)	Lower graft failure rate at 30 days, 1- and 3-years during day time. Insignificant graft failure rates in multi-variable Cox regression model.	Holiday N (25)
HCC Liver	Single,n = 147	Day (8AM-2PM) vs Night (8PM-2AM)	Increased complications including intraoperative hemorrhage, postoperative abdominal infection, and longer restoration to normal hepatic function during night.	Ren SS (30)
Kidney	Single,n = 443	Day (8AM-8PM) vs Night (8PM-8AM)	Insignificant difference in survival at 1 year.	Guo QH (21)
	Single,n = 260	Day (8AM-8PM) vs Night (8PM-8AM)	Worsen graft survival and higher re-operation at 10 years as well as higher vascular complication post-op. at night.	Fechner G (20)
	Single,n = 384	Day (6AM-11PM vs Night(11PM-6AM)	Insignificant difference in peri-operative complications and graft functions	Fockens MM (31)
	Single,n = 4515	Day (8AM-8PM) vs Night (8PM-8AM)	Insignificant difference in long-term survival, beneficial surgical technical outcomes during night time.	Brunschot DMDÖ van (26)
	Single,n = 633	Day (6AM-6PM) vs Night (6PM-6AM)	Insignificant difference in short-term survival, lower vascular complications at night time	Shaw TM (27)
	Single,n = 873	Day (8AM-8PM) vs Night (8PM-8AM)	Insignificant difference in short- and long-term graft survival (1 and 5 years). No differences in surgical complications.	Kienzl-Wagner K (28)
	Single,n = 77	Day (8AM-8PM) vs Night (8PM-8AM)	Insignificant difference in survival and peri-operative outcomes.	Guerrero ER (29)

UNOS, United Network of Organ Sharing; PGD, Primary Graft Dysfunction; HCC, Hepatocellular Carcinoma; EKiTE, Epidemiology in Kidney Transplantation.

### 2.1 Impact of donor graft retrieval time

Organ donation occurs following stringent evaluation and selection criteria after a potential donor is declared brain dead. Subsequently, graft retrieval surgery is performed by experienced harvesting surgeons to procure different organs with obtained consent authorization. During this process, careful donor management is essential to ensure stable hemodynamics and sufficient organ perfusion. Consequently, organ retrieval can occur at any time of the day to prevent graft loss due to donor deterioration. Three studies have focused on the timing of donor graft aorta cross-clamping during organ retrieval and its impact on transplant outcomes (10–12). In a study utilizing the Epidemiology in Kidney Transplantation (EKiTE) database, donor aorta clamping between midnight-to-noon (AM) compared to noon-to-midnight (PM) was associated with superior long-term survival (>5 years) (12). This finding was consistent with a study from the United Network for Organ Sharing (UNOS) heart registry (10). In another single-center heart transplant study, with an hour-dependent increase in all-cause mortality when heart grafts were harvested between 5 PM-10 PM (11). It was demonstrated that there were more than doubled death rates after propensity score matching (PSM) and increased rejection rates when harvesting during PM. These studies primarily hypothesize a circadian-regulated immunogenicity of grafts.

### 2.2 Impact of graft reperfusion (de-clamping) time

Following retrieval, most grafts undergo cold or warm ischemia depending on the preservation strategy employed. Cold preservation using solutions at 4°C on ice remains the most widely used technique due to its cost-effectiveness and efficacy. Prolonged cold ischemia can lead to more severe ischemic-reperfusion injury (IRI), resulting in early graft dysfunction. Previous evidence suggests that the timing of reperfusion within a day can influence clinical outcomes (32). Analysis using the French Registry of Organ Transplantation database (Cristal registry) demonstrated superior 1-3 year graft survival for kidneys de-clamped during 6 PM-10 AM compared to 10 AM-6 PM (13). Similarly, lungs reperfused during the high-risk window (4 AM to 8 AM) showed a significantly increased risk of primary graft dysfunction (14).

### 2.3 Impact of surgery start time

As organ donation can occur at any time of the day, the timing of surgery initiation, or “first skin incision,” has been extensively studied to determine whether after-hour effects influence transplant outcomes. These studies hypothesized that surgeon’s circadian rhythms during after-hours transplants might contribute to increased medical errors and/or reduced care quality. Notably, only 3 of 18 studies reported significant differences in graft survival (15, 19, 20). Analysis from the United Network for Organ Sharing (UNOS) registry indicated that lung transplants performed at nighttime (7 PM-7 AM vs. 7 AM-7 PM) were associated with increased mortality at 90 days, but the difference was insignificant for post-operative complications and mortality at 1 year (15). However, this finding was inconsistent with another single-center cohort study of lung transplants (6 PM-5 AM vs. 5 AM-6 PM), which showed a higher risk of major post-operative adverse events, decreased 5-year survival, and a lower bronchiolitis obliterans syndrome-free rate (19). In kidney transplants, nighttime surgeries (8 PM-8 AM vs. 8 AM-8 PM) were associated with worsened graft survival at 1 year and 10 years in two different cohorts (20, 21), and were also associated with higher post-operative vascular complications and re-operation rates in one of the studies (21). No significant differences were reported in heart and liver transplants (15–18, 25).

### 2.4 Conflicting results and the utility of studying the circadian immune system

Clinical studies examining the impact of circadian rhythms on organ transplantation outcomes have produced conflicting results. For example, some studies suggest that donor graft retrieval and reperfusion during specific times of day, such as the morning, are associated with better long-term graft survival, while others report no significant differences or even worse outcomes during certain periods. Similarly, the timing of surgery initiation has shown mixed results, with some studies indicating worse graft survival for nighttime surgeries, while others find no significant impact. These inconsistencies highlight the complexity of the relationship between circadian rhythms and transplant outcomes.

To address these conflicting results, we propose shifting the focus from merely reconciling these discrepancies to understanding the underlying mechanisms driving these variations. Specifically, we advocate for a deeper exploration of the circadian immune system, which offers a more comprehensive framework for interpreting the observed inconsistencies. By investigating the circadian regulation of immune responses, we aim to elucidate how the body’s natural rhythms can modulate the immune system’s reaction to transplanted organs. This knowledge is essential for developing evidence-based strategies that optimize graft acceptance and minimize rejection. For example, understanding the circadian variations in immune cell activity and cytokine production could inform the timing of immunosuppressive therapies to enhance their efficacy. Identifying the optimal timing for transplantation procedures based on circadian principles could also lead to improved graft survival and reduced post-transplant complications. In essence, studying the circadian immune system in transplantation is not just about resolving conflicting clinical findings; it is about uncovering fundamental biological principles that can be harnessed to improve patient outcomes.

## 3 The circadian rhythm and immune system

### 3.1 Molecular circadian clock

The circadian system encompasses a central pacemaker located in the hypothalamic suprachiasmatic nucleus (SCN) and peripheral oscillators distributed throughout all organs and tissues (5). Most organisms on Earth synchronize their circadian clocks to the 24-hour solar day, with sunrise occurring around 6 AM and sunset around 6 PM across most regions. In experimental models, Zeitgeber time (ZT) serves as the standard unit of time, representing entrained time according to the light-dark cycle, with ZT0 marking the beginning of the day (light) and ZT12 marking the beginning of the night (dark). Once entrained, organisms can be isolated from environmental cues to study the endogenous free-running period of their rhythms, measured by Circadian time (CT), where CT0 marks the start of the subjective day and CT12 marks the start of the subjective night (5).

Within each cell, autonomous transcription-translation autoregulatory feedback loops form the molecular clock, generating oscillations with an almost 24-hour periodicity. Key molecular clock genes include the transcriptional activators CLOCK and BMAL1, as well as the repressor proteins known as Period (PER1, PER2, and PER3) and Cryptochrome (CRY1 and CRY2) (33). Six stages of the mammalian circadian cycle have been established based on the molecular transcription architecture (33). These stages, ranging from the poised state to the transition back into a poised state, coincide with the regulation of cell and tissue-specific functions, ultimately driving daily cyclic variations in behavioral activities such as the sleep-wake cycle, as well as systemic functions including cardiovascular, endocrine, metabolic, nervous, and digestive functions.

### 3.2 Circadian immune system

In recent years, advancements in understanding the circadian immune system have propelled the field of therapeutic chrono-immunology, influencing strategies such as timely delivery of vaccinations, chemotherapy, bone marrow transplantation, and treatment of diseases such as asthma, neurodegeneration, and arthritis (6). It is now recognized that the entire immune system, including innate, adaptive, and gut immunity, is regulated by the circadian clock to maintain homeostasis and execute specific immune responses in a time-dependent manner.

Various aspects of the immune system, including cellular composition, development, trafficking, and response to insults, are regulated by the circadian clock. The oscillating movement of leukocyte numbers between blood, lymphoid tissues (both peaking during the behavioral rest phase), and organ tissues occurs in a circadian manner under normal conditions (34). This rhythmic variation in leukocyte numbers is governed by circadian clock-regulated mechanisms, which control leukocyte trafficking through mobilization from the bone marrow into the blood, drainage from tissues via the lymphatic system, and homing to tissues through rhythmically expressed endothelial adhesion molecules and neutrophil clearance (34–36). Response to high doses of lipopolysaccharide (LPS), bacteria and viruses also differs at distinct time of day, resulting in more fatal conditions around the start of behavioral active phase in mice; this is associated with the circadian-mobilization of Ly6C^hi^ monocytes, peaking between ZT0 (peak) and ZT12 (nadir) in blood and spleen (reciprocally in bone marrow) (37).

In the context of transplantation, the timing of bone marrow transplantation (BMT) into lethally irradiated mice has been shown to influence graft survival (38). Although not extensively explored in the field of organ transplantation, it is reasonable to speculate that allo-recognition of grafts by recipients is influenced by the time-of-day-dependent circadian-regulated immune system. A deeper understanding of the role of the circadian immune system in organ transplantation could uncover previously unidentified mechanisms and potentially lead to modifications in clinical practice to enhance graft durability.

## 4 Role of circadian-regulated passenger leukocytes in solid organ transplantation

Passenger leukocytes including antigen presenting cells and tissue-resident lymphocytes, encompass any immune cell components residing in the donor allograft and transferred into the recipient during transplantation. Past evidence has indicated that while circulating leukocytes are highly immunogenic, they are also pivotal in inducing graft tolerance (39, 40). This duality can be attributed to the fact that during transplantation, a highly heterogeneous population consisting of distinct cell types and states is transferred, depending on the organ. Furthermore, the composition, number, and states of immune cells residing within different organs also vary under different time and conditions (41).

### 4.1 Circadian-regulated antigen presenting cells in solid organ transplantation

Solid organ transplantation between genetically distinct individuals elicits an allo-reactive T cell response, which orchestrates cell-mediated transplant rejection (42). The activation of allo-reactive T cells relies on direct, indirect and semi-direct allo-recognition pathways mediated by antigen presenting cell (APC) (43). Nonetheless, the potency of the anti-donor response against the allograft depends on the frequency and/or number of host T cells that directly recognize allo-antigens, as well as the degree of host-derived infiltrated monocytes maturing into dendritic cells (DCs) indirectly (44). In the context of circadian biology, the rhythmic regulation of immune processes could significantly impact allo-recognition and subsequent rejection responses in transplantation. Hence, this section will explore how the circadian-regulated APC influencing the dynamics of allo-reactive T cell activation.

Donor-derived antigen-presenting cells are a major determinant of organ immunogenicity, influenced by the frequency of APCs with intact MHC molecules and their processing and/or differentiating stages during transplantation. Circadian regulation of circulating leukocyte APCs may contribute to time-of-day differences in graft survival. Under normal circumstances, professional antigen-presenting cells, including monocytes/macrophages, DCs, and B cells, exhibit rhythmic expression of intrinsic clock genes (BMAL1, CLOCK, PER1, and PER2), which can be entrained under conventional light-dark cycles (45). This suggests that the vast biological processes of these cells may be internally regulated by the central circadian machinery. Approximately 8% of genes in peritoneal macrophages are modulated by the circadian clock, covering several functional aspects, including phagocytosis, antigen presentation, immune regulation, and wound healing (46).

Subsequent studies have demonstrated that circadian clock-regulated antigen processing of DCs influences T cell responses (9, 47). Specifically, the antigen processing of DCs has been correlated with their mitochondrial BMAL1-dependent fusion-fission morphology. Clinically, greater T cell responses have been observed when mice are vaccinated during the middle of their rest phase compared to their active phase, coinciding with the highest DC antigen processing. This is consistent with the time-dependent efficacy of vaccines, which have been shown to be more effective when administered in the early morning compared to the afternoon (48, 49). Moreover, the initial time-of-day tumor engraftment has been shown to dictate tumor size in mouse cancer models (50). This phenomenon is attributed to circadian anti-tumor functions governed by both DCs and CD8^+^ T cells. Notably, the expression of co-stimulatory molecule CD80 by DCs, critical for generating tumor-antigen-specific CD8^+^ T cells, follows a circadian pattern, contributing to the time-of-day dosage-dependent tumor-killing potential. Fortin et al. demonstrated that circadian control of tumor immunosuppression significantly impacts the efficacy of immune checkpoint blockade in colorectal cancer (51). Key mechanisms include the circadian regulation of cytokine production, such as IL-2 and IFN-γ, which are essential for T cell proliferation and activation, as well as the influence of circadian rhythms on immune cell trafficking and the suppressive activity of regulatory T cells (Tregs) that secrete inhibitory cytokines like IL-10 and TGF-β. Additionally, the timing of administration of immune checkpoint inhibitors, which target molecules like PD-1 and CTLA-4, is crucial due to the circadian control of immune function. By understanding these circadian mechanisms, it may be possible to enhance the efficacy of immunosuppressive therapies in transplantation, potentially reducing graft rejection and improving patient outcomes. In clinical retrospective studies, differing outcomes have been observed when organs were harvested at distinct times of the day. Bioinformatics analyses have indicated a correlation between the expression of circadian regulators and pathways involving antigen processing and presentation in the heart (11).

Furthermore, after transplantation, donor-derived DCs (and other APC subsets) are required to migrate via graft-draining afferent lymphoid tissues to present donor MHC molecules directly to alloreactive T cells. This migration process is pivotal in driving early responses toward the graft by acute rejection, decreasing over time as donor circulating leukocytes deplete. Interestingly, the migration of DCs from the skin to lymph nodes via afferent lymphatic vessels occurs in a circadian manner (52). Various subsets of DCs preferentially migrate from the skin to lymph nodes during the rest phase of mice, regulated by the intrinsic circadian clock BMAL1 in both lymphatic endothelial cells and DCs. This migration pattern is determined by rhythmic gradients in the expression of the chemokine CCL21 and of adhesion molecules (e.g., ICAM-1 and VCAM-1).

Thus, it is speculated that mechanisms involving the circadian immune system may regulate graft immunogenicity, impacting allograft rejection according to the time of organ collection. To validate these mechanisms in transplant allo-recognition, future studies could employ murine models of heart or kidney transplantation, where donor organs are procured at different times of the day (53). Additionally, *in vitro* co-culture systems of donor-derived immune cells and recipient T cells could be used to assess time-of-day-dependent alloimmune responses. These approaches would help elucidate the role of circadian rhythms in graft outcomes.

### 4.2 Circadian-regulated subsets of passenger lymphocytes and mechanism implicated in solid organ transplantation

Passenger Lymphocytes, in addition to the APC subsets, can also play a crucial role in priming the recipient’s adaptive allo-immunity or inducing tolerance (54). A diverse population of passenger lymphocytes has been previously studied in solid organ transplantation, each with post-transplant functional specificity. Passenger T lymphocytes including CD4^+^ T helper cell (T_H_) subsets such as T_H_1, T_H_2, T_H_17, T regulatory (T_reg_), as well as follicular T helper (T_FH_) cells and CD8^+^ T cells identified by their memory and effector functions. Organs such as the lung, liver, kidney and gut contain abundant non-migratory, tissue-resident innate-like lymphoid cells (ILC) including ILC1, ILC2 and ILC3 and tissue-resident memory T cells (T_RM_) (55, 56). After organ transplantation, these passenger lymphocytes may remain and proliferate in the graft, emigrate from the graft site, or be eliminated by acute rejection, while participating in the activation of the recipient allo-immunity or inducing tolerance.

Donor CD4^+^ T cells that evade host NK cell missing-self killing mechanism can activate the recipient’s adaptive allo-immunity and signal B cells to produce autoantibodies, leading to accelerated allograft vasculopathy (antibody-mediated rejection, AMR) (57–60). This phenomenon was further elucidated in a recent study utilizing a CD3e knockout recipient in a murine model of allogeneic heart transplantation (60). Despite lacking T cells, recipient mice exhibited a rapid, transient wave of switched alloantibodies predominantly directed against MHC I molecules (donor-specific antibodies, DSA) after transplantation, solely induced by donor T cells, thus delineating an inverted direct allo-recognition pathway.

Conversely, in another murine model of cardiac allograft vasculopathy, depletion of donor CD4^+^ T_reg_ (FOXP3^+^ CD4^+^) before organ recovery resulted in markedly accelerated heart allograft rejection and augmented host effector antibody responses (61). Adoptive transfer of purified donor-strain natural T_reg_ inhibited host humoral immunity, prolonged allograft survival, and did so more effectively than recipient T_reg_.

Current evidence suggests the presence of intrinsic molecular circadian clocks in both CD4^+^ and CD8^+^ subsets, regulating various functional aspects such as activation, proliferation, and differentiation capacity (9). Importantly, core clock genes participate in the differentiation of CD4^+^ T cells into specialized subsets such as T_reg_ or T_H_17 (62, 63). However, it remains unclear whether these clock genes play a role in the time-of-day effects observed in organ transplantation. Further investigations are warranted to elucidate the potential involvement of circadian rhythms in modulating the behavior of donor lymphocytes and their impact on allorecognition and graft outcomes.

Innate lymphoid cell (ILC) represents a population of innate immune cells residing in tissue with morphology and functions resembling the CD4^+^ T cells but lack rearranged antigen receptors (64). Thus, ILC do not possess cytotoxic function and instead exert their functionality through robust production of cytokines and growth factors. The functional role of ILC as passenger during solid organ transplantations were not extensively studied. However, a study has shown that the proportion of ILC before and after lung transplant correlates with the occurrence of primary graft dysfunction (PGD) (65). In the study, it was found that patients without PGD exhibited significantly higher ILC1 frequencies before reperfusion, whereas the percentage of group 2 ILC (ILC2) were selectively increased after allograft perfusion, suggesting that these cells may protect against PGD. It was noted that ILC2 and ILC3 expressed high levels of circadian genes and have demonstrated an intrinsic circadian clock that can be entrained by light and are essential for maintaining immunity, and nutrient signaling and intestinal gut homeostasis (56, 66–68). The number of ILC3 cells present in the small intestinal lamina propria fluctuates according to the behavioral phases of organisms, accumulating and becoming more activated during the rest phase compared to the active phase. Similarly, graft ILCs have been replaced by substantial recipient ILCs in both the epithelium and lamina propria. However, the circadian control of ILC and their impact in solid organ transplantation remain largely unknown.

Tissue-resident memory T cells (T_RM_) are generated and retained in non-lymphoid tissues following site-specific infection or antigen exposure, playing a crucial role in local secondary defense through immediate cytotoxic response as well as recruitment and activation of other defense responders (69). Consequently, they are more abundant in grafts such as lungs, liver, and intestines (70). During organ transplantation, T_RM_ cells have been shown to contribute to post-transplant outcomes. Donor T_RM_ that migrate out of the graft can be detected in the blood for a limited time (months to years), potentially contributing to graft-versus-host disease (GvHD). Host effector T cells subsequently reject donor-derived passenger cells in both the blood and retaining tissue cells, ultimately leading to the complete replacement of the lymphocyte population. Conversely, higher persisting donor T cells have been associated with clinically protective outcomes in lung and intestinal transplantation, as evidenced by reduced rejection rates and primary graft dysfunction in lungs (55, 71). Grafts with a higher population of T_RM_ have exhibited the lowest long-term graft survival compared to other organs such as the heart and kidney, although the underlying mechanisms remain incompletely understood. Current understanding suggests that following transplantation, distinct subsets of TRM cells may either migrate out of or be retained within the transplanted allograft. However, whether circadian rhythms impact transplant outcomes such as chimerism or GvHD through T_RM_ remains unknown.

The intrinsic circadian clock of B lymphocytes has been less studied in the context of organ transplantation due to their low frequency in grafts (45). However, the immune system exhibits daily variations in cellular composition, trafficking, and responses to injury. For example, donor passenger leukocytes, such as dendritic cells and macrophages, may exhibit time-of-day-dependent changes in activation states or cytokine profiles, potentially influencing graft outcomes (see **Table 2** for details). However, further studies are needed to uncover the potential of harnessing the circadian clock to enhance graft durability in the future.

**Table 2:** Circadian rhythm of immune cell subsets and the role implicated in solid organ transplantation.

Cell Types/Events	Related Circadian Functions	Discovered Mechanisms	Possible Role in Transplantation	Ref.
Neutrophil	- Recruitment to sites of tissue damage or infection is time-of-day dependent.	- Driven by expression of adhesion molecules (e.g., *ICAM-1* and *VCAM-1*) and chemokines in endothelial cells	- Higher neutrophil activity during certain times might lead to increased T-cell activation and graft rejection.	(72)
Monocyte/Macrophage	- Phagocytic activity and cytokine production peak during the active phase.	- Clock genes (e.g., *BMAL1*, *PER2*) regulate monocyte activation and infiltration.	- Direct/indirect allo-recognition involving donor/recipient monocyte cells leading to acute/chronic rejection may be time-of-day dependent.	(37, 73)
Dendritic Cells (DCs)	- Antigen-specific CD8+ T cell expansion is time-of-day dependent (higher during rest phase). - Migration to lymph nodes peaks during the middle of the rest phase and troughs during the active phase.	- DC intrinsic clock regulates co-stimulatory molecule expression (e.g., *CD80*) and antigen presentation.	- Direct allo-recognition leading to acute cellular rejection may vary with the time of day.	(47, 50, 52)
CD4^+^ T Cells	- Lymphocyte counts in blood and lymph peak during the rest phase, while tissue counts peak at the onset of the active phase.	- Circadian regulation of T cell trafficking and activation is mediated by clock genes (e.g., *REV-ERBα*).	- Inverted regulation leading to allo-antibody production may be time-of-day dependent.	(60)
CD8^+^ T cells	-Cytotoxic activity and cytokine production peaking during the active phase of the day-night cycle.	-Expression of immune checkpoint gene *PDCD1* (PD-1) showed rhythmic expression and can be abrogated by *Bmal1*, *Per1* or *Per2* deletion.	- Timing of immune interventions could enhance their efficacy in transplantation settings.	(74)
T Helper 17 (TH17)	- Naive T cells isolated during the day are more likely to differentiate into TH17 (regulated by *Nr1d1*, *Rorc*, *Nfil3*).	- Clock genes influence TH17 differentiation and cytokine production.	- Absence of donor TH17 exacerbates graft-versus-host disease (GvHD).	(62)
Regulatory T Cells (Treg)	- Circadian regulation of Treg function is not fully understood.	- Tregs exhibit rhythmic expression of clock genes, influencing immune tolerance.	- Absence of donor Tregs decreases graft tolerance.	(63)
Tissue-Resident Memory T Cells (TRM)/Innate Lymphoid Cells (ILC)	- ILC3 numbers and functions fluctuate (lower/non-activated during active phase, higher/active during rest phase).	- Circadian regulation of ILC3 influences tissue homeostasis and immune responses.	- Donor TRM exiting the graft into blood mediates GvHD, while persistent TRM in the graft reduces rejection.	(55, 71)
B Lymphocytes	- Dispensable for circadian regulation but involved in differentiation and maturation.	- B cell intrinsic clock regulates antigen presentation and antibody production.	- May act as professional antigen-presenting cells, influencing direct/indirect allo-recognition.	(45)
Regulated cell death (RCD)	- Several clock genes determine fate and mode of RCD during homeostasis and disease	- Clock genes such as BMAL1 and CLOCK play a role in regulating cell death through autophagy and mitochondrial clearance.	-Mode of RCD determines immunogenicity of DAMPs for indirect allo-recognition.	(75)

DCs, Dendritic cells; T_H_17, T helper-17; T_reg_, Regulatory-T; T_RM_, Tissue-resident memory T cell; ILC, Innate lymphocyte cell; GvHD, Graft-versus host disease; IRI, Ischemia-reperfusion injury; RCD, regulated cell death.

## 5 Role of circadian-regulated cell death and immune response in solid organ transplant

### 5.1 Cold ischemia-reperfusion injury and tissue tolerance regulated by circadian rhythm

Organ preservation in cold preservative solutions provides protective effects by reducing cellular metabolism and maintaining osmotic pressure. However, prolonged cold ischemia in various organs can lead to increased accumulation of succinate, which upon reperfusion is rapidly re-oxidized by succinate dehydrogenase, generating extensive reactive oxidative species (ROS) through reverse electron transport at mitochondrial complex I (76, 77). It has been firmly established that tissue tolerance to IRI, especially in myocardial tissue, is strikingly time-dependent and regulated by the circadian clock (32, 75, 78, 79).

This notion is supported by several core clock genes, including *PER2*, which regulate oxygen sensing and metabolism within cells to mitigate the impact of ROS bursts during reperfusion (78, 79). Numerous potential therapeutic strategies exist to modify clock gene expression to enhance tissue tolerance to IRI (80, 81). These strategies include interventions targeting environmental cues, such as intense light therapy, carbon monoxide inhalation (82, 83), as well as pharmacological interventions with purinergic receptor agonists, ADORAB2 agonists, and REV-ERB agonists directly targeting clock genes (32, 79).

### 5.2 Severe ischemia-reperfusion injury and circadian-regulated cell death mechanisms

Severe IRI can lead to cell death, resulting in graft dysfunction due to the loss of functional cells such as tubular cells in the kidney, cardiomyocytes in the heart, and hepatocytes in the liver. Clinically, organs with higher metabolic demands, such as the heart, typically have a shorter cold ischemic time window (< 4 hours) compared to other organs like the liver or kidney (84, 85). Furthermore, the loss of cardiomyocytes can lead to irreversible injury due to their limited regenerative capacity, underscoring the importance of cardio-protection in the recovery and preservation of donor hearts (86).

Traditionally, necrosis and apoptosis have been considered the central hallmark mechanisms of IRI, with necrosis viewed as an “accidental” form of cell death and apoptosis as a programmed “suicidal” form (87). However, recent research has unveiled various subtypes of apoptosis (intrinsic, extrinsic) and regulated necrosis, as well as several novel forms of regulated cell death (RCD), including ferroptosis, necroptosis, autophagy-dependent cell death, and pyroptosis. These forms of cell death have been named according to their hallmark features and regulatory mechanisms (88–91).

Emerging evidence is gradually uncovering the roles of distinct cell death mechanisms, their occurrence stages, and consequences in transplant IRI (91–95). For example, ferroptosis can be triggered during cold stress due to upregulated mitochondrial calcium uptake regulator (MICU1) and endoplasmic reticulum stress (96), while pyroptosis is mainly described in myeloid cells, enhancing the secretion of inflammasomes and interleukins such as IL-1β and IL-18 (95, 97–99). Interestingly, circadian clock genes participate in regulating cell fates through apoptosis, necrosis, autophagy, ferroptosis, and many other forms of cell death (75, 100–102). This highlights the intricate interplay between circadian rhythms, cell death mechanisms and the activation of immune cells, suggesting potential avenues for therapeutic interventions to mitigate the detrimental effects of IRI in organ transplantation.

### 5.3 The role of DAMPs mediating circadian-regulated outcome in IRI

The release of DAMPs from stressed or dying cells represents a major source of immunogens that promote innate and adaptive immune responses (103). Several members of the DAMPs implicated in organ transplantation, such as high mobility group box 1 (HMGB1), extracellular adenosine triphosphate (eATP), and major histocompatibility complex class I chain-related proteins A and B (MICA, MICB), have been proposed (73). These DAMPs can be actively secreted by damaged allograft cells and taken up by both donor- and recipient-derived DCs, promoting immunostimulatory maturation that activates Th17 and Th1 alloreactive T cells.

While it is reasonable to speculate that time-of-day-dependent injury during IRI may result in a dose-dependent release of DAMPs mediating macrophage and/or DC maturation to enhance allograft rejection, many questions remain unexplored. For example, it was previously understood that immunogenic cell death (ICD) occurring in different scenarios may activate distinct DAMP-induced pathways and immune responses (104). Additionally, it is known that apoptotic cells that are rapidly phagocytosed can escape triggering an immune response. However, other modes of apoptosis and regulated cell deaths, such as necrosis, necroptosis, and autophagy, possess specific immunomodulatory functions, as extensively reviewed in the field of cancer (104).

However, whether time-of-day transplant IRI results in distinct forms of regulated cell death and their subsequent prognosis remains largely unexplored. Understanding the interplay between circadian rhythms, mode of regulated cell death, and the release of DAMPs could provide valuable insights into the mechanisms underlying allograft rejection and identify potential targets for therapeutic intervention in organ transplantation.

### 5.4 Circadian-regulated immune cell recruitment and inflammation in IRI

Neutrophils and monocytes/macrophages play pivotal roles in ischemia-reperfusion injury (IRI) by clearing dead cells and contributing to the inflammatory response. Recent studies reveal that these immune cells exhibit distinct responses based on their circadian rhythms, with implications for the severity of tissue damage. For instance, Sun et al. demonstrated that neutrophil infiltration in renal IRI follows a circadian pattern, suggesting that timing relative to the body’s internal clock influences the extent of damage (105). Neutrophil extracellular traps (NETs) and neutrophil-derived cytokines contribute to tissue damage during reperfusion. Given the circadian rhythmicity of neutrophil function, the timing of organ procurement and reperfusion could influence the severity of IRI (72). For example, neutrophil recruitment and NET formation might be more pronounced during certain times of the day, potentially exacerbating graft injury. Neutrophils are involved in both acute and chronic graft rejection (106). They can secrete T-cell chemo-attractants and cross-prime T cells, enhancing alloimmune responses. The circadian rhythm of neutrophils could affect their ability to initiate and propagate rejection. For instance, higher neutrophil activity during certain times might lead to increased T-cell activation and graft rejection.

Similarly, monocytes exhibit circadian rhythmicity in their functions, including migration, cytokine production, and phagocytic activity (37). The core circadian clock genes, such as BMAL1 and CLOCK, regulate the expression of clock-controlled genes that influence monocyte behavior. Monocytes show rhythmic expression of pro-inflammatory cytokines such as IL-6 and TNF-α, with peak expression occurring at specific times of the day. This rhythmicity is influenced by the circadian clock. Monocytes play a crucial role in the immune response following solid organ transplantation, particularly in IRI and graft rejection. Monocytes are rapidly recruited to the site of ischemia-reperfusion, contributing to tissue damage (73). The timing of organ procurement and reperfusion can influence the severity of IRI due to the circadian rhythmicity of monocyte recruitment and activation. Monocytes can differentiate into macrophages and dendritic cells, which are key players in initiating and propagating alloimmune responses. Dong et al. found that macrophage activation occurs in a time-dependent manner, highlighting the potential modulation of immune cell activity by circadian rhythms (107). The circadian rhythm of monocytes can affect their ability to activate T cells and contribute to graft rejection. These findings underscore the potential therapeutic value of manipulating tissue circadian rhythms to mitigate IRI-associated damage in organ transplants. Understanding and modulating these cyclic patterns could offer novel strategies for improving post-transplant outcomes and reducing complications.

Emerging research on CD8^+^ T cells further underscores the importance of circadian regulation in immune responses. A recent study published revealed that CD8+ T cell function is tightly regulated by circadian rhythms, with their cytotoxic activity and cytokine production peaking during the active phase of the day-night cycle (74). This rhythmicity is driven by intrinsic clock genes, which modulate metabolic pathways and effector functions in CD8+ T cells. Importantly, the study demonstrated that disrupting these circadian rhythms impairs CD8+ T cell-mediated immunity, suggesting that optimal timing of immune interventions could enhance their efficacy in transplantation settings.

These findings collectively underscore the therapeutic potential of leveraging circadian rhythms to modulate immune cell activity and mitigate IRI-associated damage in organ transplants. By understanding and manipulating these cyclic patterns, clinicians and researchers could develop novel strategies to improve post-transplant outcomes, reduce complications, and optimize immunosuppressive therapies. Future studies should explore the interplay between donor and recipient circadian rhythms, as well as the impact of circadian disruption on long-term graft survival.

## 6 Guidelines for studying circadian rhythms in transplant immunology

Circadian rhythms—the endogenous 24-hour cycles that regulate physiological processes—play a pivotal role in immune function and transplant outcomes. Disruptions in these rhythms, whether due to organ procurement timing, recipient lifestyle, or mismatched donor-recipient cycles, can influence graft rejection, immunosuppressant pharmacokinetics, and inflammatory responses. To systematically investigate circadian interactions in transplantation, researchers must adopt standardized methodologies that account for temporal variability. Below, we outline best practices for designing and interpreting circadian-focused studies in transplant immunology, emphasizing reproducibility, mechanistic clarity, and translational relevance (see **Table 3**).

**Table 3:** Best practices for circadian transplant immunology studies.

Category	Recommendations
Sample collection	Standardize organ procurement to a 2–4-hour window; document donor/recipient chronotype using melatonin assays or actigraphy.
Animal models	House rodents under 12:12-hour light-dark cycles ≥2 weeks pre-study; use Bmal1^-^/^-^ mice to test clock gene mechanisms.
*In-vitro* systems	Synchronize immune cells via serum shock or temperature cycles; assay functions across 24-hour periods.
Data analysis	Apply cosinor or circular statistics; adjust for time-dependent confounders (e.g., drug dosing schedules).
Clinical Translation	Stratify patients by chronotype; monitor circadian biomarkers (e.g., cortisol) pre-/post-transplant.

### 6.1 Timing and sample collection

The optimal time for transplantation should align with the peak activity of the donor’s/recipient’s immune system. Standardizing sample collection within a 2-4-hour window during specific circadian phases (such as peak activity phases in the morning or evening) can help capture consistent data, ensuring that biological measurements reflect individualized rhythms rather than artificial or arbitrary timings. The circadian rhythm’s distinct phases suggest that individuals may have different peaks (e.g., some are most active in the morning, others in the evening), and a 2-4-hour window aligns with these key periods. This synchronization helps standardize data collection, making it easier to compare across individuals by reducing variability from external factors. The specific determination of this window likely involves empirical testing or reference to existing circadian rhythm data that identifies optimal timing for minimizing variability (108). Documentation methods, such as measuring melatonin levels or assessing sleep-wake cycles, can also provide valuable insights into individual circadian alignment, aiding in optimizing transplant timing.

### 6.2 Experimental models

Animal models, such as rodents under controlled light-dark cycles, offer a controlled environment for studying the effects of circadian rhythms on immune responses. Conditional genetic disruptions of core clock genes (e.g., *Bmal1*, *Per2 and Nr1d1/Nr1d2*) can reveal mechanistic links between circadian rhythms and immune function (109). Additionally, ex vivo systems using perfused donor organs allow isolation of tissue-specific clocks, which may differ from systemic rhythms, providing unique insights into organ-level regulation.

### 6.3 Data analysis

Advanced statistical approaches, such as cosinor analysis and mixed-effects models, are employed to identify periodic patterns in biological data and account for inter-individual variability. Replicating findings across multiple circadian cycles and validating them in different species enhances the robustness of research outcomes. This systematic approach ensures that conclusions are grounded in reliable and reproducible evidence.

### 6.4 Additional methodological considerations

In clinical trials, stratifying transplant donors and/or recipients by chronotype—assessed via validated questionnaires such as the Munich Chronotype Questionnaire—can reveal subgroups with differential susceptibility to circadian disruption (110, 111). Wearable devices (e.g., actigraphy watches) enable continuous monitoring of rest-activity cycles in patients, while telemetry in animal models tracks core body temperature or locomotor activity rhythms. Emerging technologies like single-cell RNA sequencing offer high-resolution insights into circadian gene expression within immune cell subsets. Transparent reporting of experimental conditions—including Zeitgeber Time (ZT), light intensity (lux), and synchronization protocols—is critical for reproducibility. Researchers should also disclose potential masking effects, such as anesthesia during nighttime organ procurement or timed feeding regimens.

In summary, circadian biology introduces a layer of temporal complexity to transplant immunology that, if ignored, risks obscuring critical determinants of graft survival and immune regulation. By rigorously standardizing sample timing, leveraging synchronized models, and applying rhythm-aware analytics, researchers can unravel circadian mechanisms with translational potential. Future work should prioritize bridging preclinical findings to clinical trials that test chronotherapeutic strategies, such as timed immunosuppressant delivery or donor-recipient cycle alignment. Adherence to these guidelines will enhance the validity of circadian research in transplantation and accelerate its integration into precision medicine frameworks.

## 7 Conclusion and future perspective

### 7.1 Limitations and future directions

While retrospective studies have provided valuable insights into the role of circadian rhythms in transplantation, several limitations must be acknowledged. First, the reliance on observational data introduces the potential for confounding by unmeasured variables. Second, experimental models often fail to fully replicate the complexity of human immune responses, limiting their translational relevance. To address these challenges, future research should prioritize prospective clinical trials and advanced *in vitro* models that better mimic human physiology. Additionally, the development of standardized guidelines for studying circadian rhythms in transplantation would enhance cross-study comparability and accelerate progress in this field.

**Figure 1:**
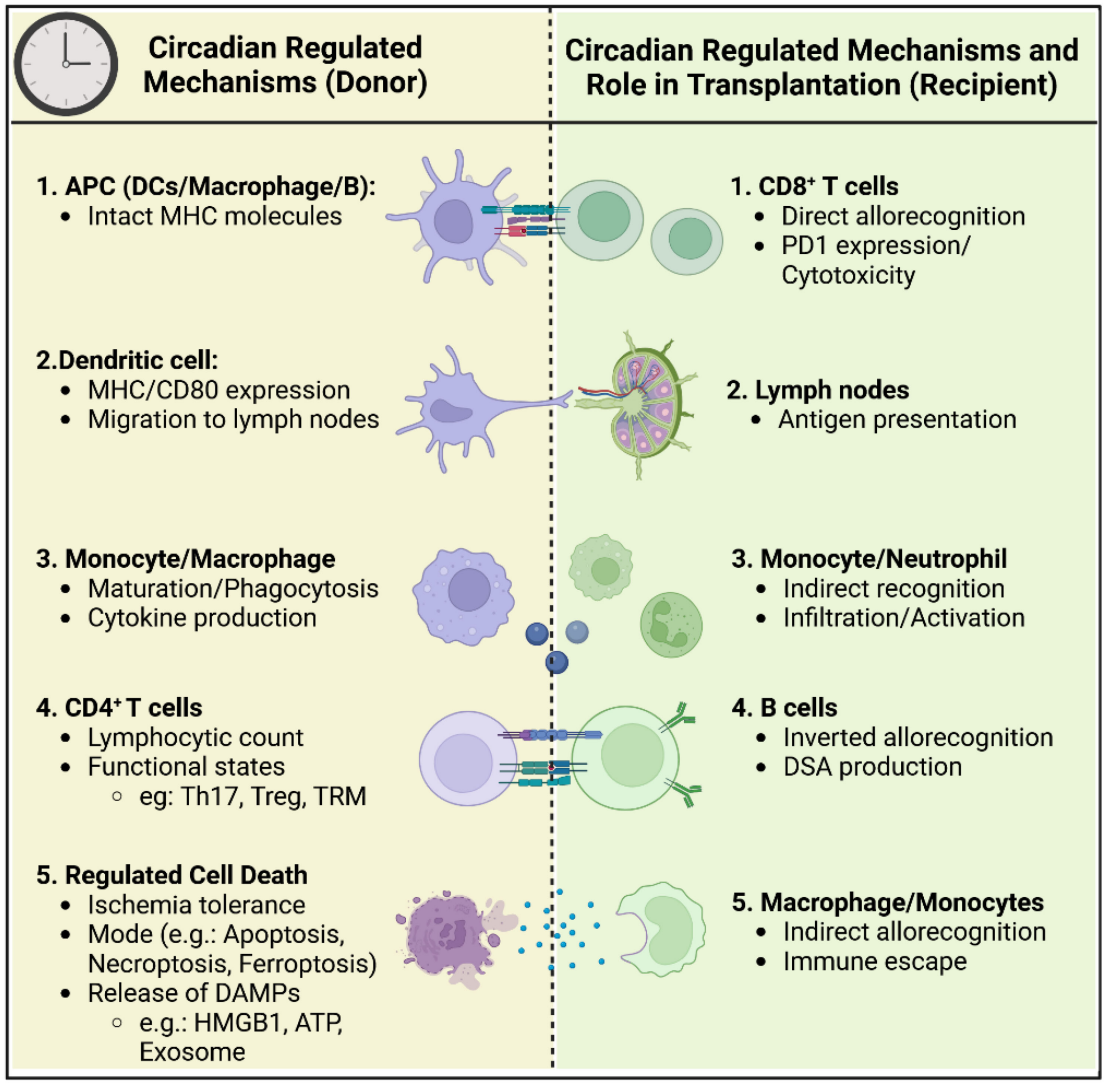
Circadian regulation of immune cells in donor and recipient during organ transplantation. This schematic figure illustrates the circadian-regulated mechanisms influencing the immune response in both the donor and recipient during the process of organ transplantation. The left side of the figure (Donor) details the circadian-regulated processes in antigen-presenting cells (APCs), dendritic cells, monocytes/macrophages, CD4+ T cells, and regulated cell death. The right side of the figure (Recipient) shows the corresponding circadian-regulated mechanisms and roles in CD8+ T cells, lymph nodes, monocytes/neutrophils, B cells, and macrophages/monocytes.

Clinical studies have consistently suggested that the timing of organ procurement and transplantation may influence clinical outcomes. However, inconsistencies in findings are largely attributed to the heterogeneity of study designs, including variations in sample sizes, organ types, and methodologies. Despite these challenges, current research has identified several key mechanisms through which circadian rhythms may impact transplantation outcomes (mechanisms summarized in **Figure 1**):

1. Circadian-Regulated Migration and Function of Circulating Leukocyte Subsets: Emerging evidence underscores the role of circadian rhythms in governing the migration behavior and functional roles of passenger leukocyte subsets, including antigen-presenting cells and lymphocytes. Understanding how these subsets are influenced by circadian rhythms could provide insights into their contribution to allograft rejection and tolerance.
2. Ischemia-Reperfusion Injury Tolerance and Mode of Cell Death: Circadian clock mechanisms play a pivotal role in regulating tolerance to ischemia-reperfusion injury and determining the mode of cell death, which subsequently leads to the release of immunogenic DAMPs. Unraveling the interplay between circadian rhythms and these processes could shed light on novel therapeutic targets for mitigating allograft rejection and improving transplant outcomes.

Despite these advances, significant gaps in knowledge remain. For instance, the relative contributions of individual circadian-regulated mechanisms to transplant outcomes are not yet fully understood. A comprehensive review of the current literature, combined with a deeper exploration of the circadian immune system, holds promise for identifying new therapeutic targets.

### 7.2 Therapeutic tool development examples

#### 7.2.1 Circadian modifiers

Developing drugs or interventions that manipulate clock genes (e.g., BMAL1, CLOCK) to optimize immune responses and reduce ischemia-reperfusion injury could enhance graft survival and patient prognosis.

#### 7.2.2 Timing optimization

Investigating whether shifting organ procurement or transplantation times aligns with the circadian rhythm of both donors and recipients to enhance graft acceptance.

#### 7.2.3 Circadian-sensitive biomarkers

Identifying biomarkers that indicate optimal timing for interventions, such as immune cell mobilization or anti-rejection therapy.

#### 7.2.4 Precision transplantation schedules

Creating personalized schedules based on individual circadian rhythms to minimize complications and improve outcomes.

Translating these findings into clinical practice will require further research, including well-designed preclinical studies and multicenter clinical trials. By addressing the identified limitations and leveraging advances in circadian rhythm research, the field has the potential to develop innovative therapeutic strategies that significantly enhance transplant success rates and patient prognoses.
